# Cardiomyopathy among first- and second-generation immigrants in Sweden: a nationwide total population study

**DOI:** 10.1186/s12872-022-02968-0

**Published:** 2022-12-06

**Authors:** Per Wändell, Xinjun Li, Axel C. Carlsson, Jan Sundquist, Kristina Sundquist

**Affiliations:** 1 Division of Family Medicine and Primary Care, Department of Neurobiology, Care Sciences and Society, Karolinska Institutet, Huddinge, Sweden; 2 Center for Primary Health Care Research, Lund University, Malmö, Sweden; 3 Academic Primary Health Care Centre, Stockholm Region, Stockholm, Sweden; 4 Department of Family Medicine and Community Health, Icahn School of Medicine at Mount Sinai, New York, NY USA; 5 Department of Population Health Science and Policy, Icahn School of Medicine at Mount Sinai, New York, NY USA; 6 Department of Functional Pathology, Center for Community-Based Healthcare Research and Education (CoHRE), School of Medicine, Shimane University, Matsue, Japan

**Keywords:** Cardiomyopathy, Immigrants, Neighborhood, Sex, Socioeconomic status

## Abstract

**Purpose:**

We aimed to analyze the risk of cardiomyopathies (CMPs) among first-generation and second-generation immigrants.

**Methods:**

All individuals aged 18 years of age and older, n = 6,123,661 in the first-generation study, and n = 4,587,764 in the second-generation study were analyzed. CMP was defined as at least one registered diagnosis in the National Patient Register between January 1, 1998 and December 31, 2018. Cox regression analysis was used to estimate the relative risk (hazard ratios (HR) with 99% confidence intervals (CI)) of incident CMP with adjustments made for age, cancer, other comorbidities, and sociodemographic factors.

**Results:**

In the first-generation study, a total of 33,321 CMP cases were registered, 20,780 men and 12,541 women, where the fully adjusted models showed HRs (99% CI) for all foreign-born men of 0.92 (0.86–0.98) and for women of 0.90 (0.83–0.98). For dilated CMP, the risk was higher for men from Nordic countries, more specifically men from Finland, and lower for men and women from Asia. For hypertrophic CMP, the risk was higher for men from Africa and Asia. For other types of CMPs, the risk was lower in men and women from Asia. In the second-generation study, a total of 26,559 cases were registered (17,620 men and 8939 women), with no significant differences overall or among specific groups, when Swedish-born with foreign-born parents were compared to Swedish-born with Swedish-born parents.

**Conclusions:**

We observed a generally lower risk of CMPs among foreign-born individuals, but with a higher risk especially for hypertrophic CMPs for men from Africa and Asia, and a higher risk of dilated CMP for men from Nordic countries.

**Supplementary Information:**

The online version contains supplementary material available at 10.1186/s12872-022-02968-0.

## Introduction

Cardiomyopathies (CMPs) are important as causes of congestive heart failure (CHF) and are crucial to identify in order to treat properly. Globally, as regards background factors for CHF, CMPs are most common in high-income Asian Pacific countries, Sub-Saharan Africa, Latin America and the Caribbean, while lower in other regions of the world [1].

CMPs can be classified in different ways, with the proposal from the European Society of Cardiology (ESC) dividing them into hypertrophic CMP (HCM), dilated CMP (DCM), arrhythmogenic right ventricular CMP (ARVC), restrictive CMP (RCM) and unclassified [2]. There are familial (genetic) dilated types [3], hypertrophic types [4], and non-familial types. Hypertrophic CMP is the most common inherited heart disease [5].

Valvular heart diseases were historically caused by rheumatic heart diseases as a consequence of infections, often by streptococci. However, acute rheumatic fever has decreased during recent decades in industrialized or developed countries, while it is still more common in other parts of the world [6].

CMP is associated with different diseases [7]. Substance abuse is associated with cardiac toxicity, mostly alcohol CMP, which has been described as one of the main causes of non-ischemic dilated CMP [8].

There are several other types of CMPs, including familial Mediterranean fever, which is an autoinflammatory disorder [9], atrial arrhythmogenic right ventricular CMP [10], or Takotsubo CMP [11], thus making it important to study first and second-generation immigrants. Furthermore, many other diseases are associated with CMPs, such as different types of metabolic diseases like amyloidosis, sarcoidosis [12], hemochromatosis, connective tissue disorders, and endocrinological diseases such as diabetes [13, 14] or thyroid disorders as well as cancer.

As there is a paucity of studies on CMP in immigrants in Sweden and other Western countries, this study will fill a knowledge gap. Enhanced knowledge of the risk of CMPs is important both for healthcare and other parts of society in Sweden as well as for other Western countries. Accordingly, our study aimed to estimate the risk of CMPs in general among foreign-born individuals in Sweden compared to Swedes and also sub-categorized as dilated, hypertrophic and other types of CMPs. We also aimed to study CMPs in second-generation immigrants and compare Swedish-born individuals with Swedish-born parents, to obtain more knowledge of the potential genetic and environmental origin of clinically observed CMPs.

## Methods

We used national Swedish registers, i.e. the Swedish National Patient Register (NPR), and the Swedish Total Population Register. The NPR includes diagnoses from all Swedish hospitals, i.e. for in-patients since 1987 and for out-patients from 2001 onwards. The Total Population Register includes data on country of origin and sociodemographic factors on all persons in Sweden with a residence permit. Our study was conducted using pseudonymized data. All methods were performed in accordance with the relevant ethical guidelines and regulations in Sweden.

### Study population

We included individuals 18 years of age and older and excluded individuals with a diagnosis of CMP before 1998, in total 5037 individuals. A total of 6,123,661 individuals were included in the first-generation study, 2,971,780 men and 3,151,881 women. In the second-generation study, a total of 4,587,764 individuals were included, 2,345,774 men and 2,241,990 women.

### Outcomes

We included the following diagnoses (with ICD-10 codes): 1. Dilated CMP (I42.0); 2. Hypertrophic CMP (I42.1–I42.2); and 3. All other types of CMPs (I42.3–I42.9, I43). We also sub-categorized patients into these three main groups. There is no national policy on response to identifying an index case of cardiomyopathy.

### Sociodemographic variables

The population was stratified by *sex*.

*Age* was used as a continuous variable in the analysis.

*Educational attainment* was categorized as ≤ 9 years (partial or complete compulsory schooling), 10–12 years (partial or complete secondary schooling) and > 12 years (attendance at college and/or university).

*Geographic region of residence* was included to adjust for possible regional differences in hospital admissions and was categorized as the following: (1) large cities, (2) southern Sweden and (3) northern Sweden. Large cities were defined as municipalities with a population of > 200,000 and comprised the three largest cities in Sweden: Stockholm, Gothenburg, and Malmö.

*Neighborhood socioeconomic levels* were derived from Small Area Market Statistics (SAMS). The average population in each SAMS neighborhood is approximately 2000 people for Stockholm and 1000 people for the rest of Sweden. A summary index was calculated to characterize neighborhood-level deprivation. The index was categorized into three groups: more than one standard deviation (SD) below the mean (high SES or low deprivation level), more than one SD above the mean (low SES or high deprivation level), and within one SD of the mean (middle SES or middle deprivation level) [15], with neighborhood status classified as high, middle, or low SES, corresponding to the categories low, middle, and high deprivation in the index [16].

### Comorbidities

We included the following comorbidities (with ICD-10 codes): hypertension (I10–I19), coronary heart disease (CHD I20–I25), chronic rheumatic heart disease (I05–I09), non-rheumatic valvular heart diseases (I34–I39), atrial fibrillation (AF I48), congestive heart disease (CHF I50, I11.0), stroke (I60–I69), diabetes mellitus (E10–E14), thyroid disorders (hypothyroidism E02–E03 and hyperthyroidism E05), chronic obstructive pulmonary disease (COPD J40–J47), alcoholism and related disorders (F10, K70), systemic connective tissue disorders (M30–M36), amyloidosis (E85), sarcoidosis (D86), hemochromatosis (E83.1), cancers (C00–C97) and Chagas disease (B57). All types of cancers (C00–C97) were also included as they represent a major cause of death in both Sweden and most other countries.

### Statistical analysis

The number of cases of CMP in the first-generation study was presented for all groups and across baseline subject characteristics. We used Cox regression analysis with Hazard Ratios (HRs) and 99% Confidence Intervals (99% CI) to estimate the risk of incident CMPs in different immigrant groups compared to the Swedish-born population, and in second-generation immigrants compared to Swedish-born individuals with Swedish-born parents. All analyzes were stratified by sex. Three models were used: Model 1 was adjusted for age and region of residence in Sweden; Model 2 was adjusted for age, region of residence in Sweden, educational level, marital status, and neighborhood SES; Model 3 was constructed as Model 2 with the inclusion of comorbidities. Analyzes were performed firstly with all CMPs included, secondly by categorizing by age ≤ 54 years of age and > 54 years, and thirdly by categorizing into dilated CMP, hypertrophic CMP, and all other types. We also added a sensitivity analysis that was adjusted for age and cancers.

In addition, we studied second-generation individuals in the same way as first-generation immigrants.

## Results

In total 6,123,661 individuals were included in the first-generation study, 2,971,780 men and 3,151,881 women, with 524,226 foreign-born men and 510,760 foreign-born women (Table 1, Additional file 1: Tables 1a and b). A total of 33,321 CMP cases were registered, 20,780 men and 12,541 women, with 2566 CMP cases among foreign-born men and 1499 among foreign-born women.

**Table 1:** The population in the first-generation study and number of cardiomyopathy (CMP) cases categorized by sex

	Men				Women			
	Population		CMPs		Population		CMPs	
	Number	%	Number	%	Number	%	Number	%
Total population	2,971,780		20,780		3,151,881		12,541	
Immigrant status								
Swedish	2,447,554	82.4	18,214	87.7	2,641,121	83.8	11,042	88.0
Foreign-born	524,226	17.6	2566	12.3	510,760	16.2	1499	12.0
Age (years)								
18–39	1,175,337	39.5	3422	16.5	1,161,867	36.9	1826	14.6
40–49	514,669	17.3	4293	20.7	527,494	16.7	2259	18.0
50–59	531,650	17.9	6333	30.5	534,858	17.0	3762	30.0
60+	750,124	25.2	6732	32.4	927,662	29.4	4694	37.4
Educational level								
≤ 9	1,124,487	37.8	8013	38.6	1,164,144	36.9	4885	39.0
10–12	1,188,546	40.0	8621	41.5	1,244,466	39.5	5117	40.8
> 12	658,747	22.2	4146	20.0	743,271	23.6	2539	20.2
Region of residence								
Large cities	1,297,742	43.7	10,431	50.2	1,413,075	44.8	6363	50.7
Southern Sweden	915,205	30.8	6605	31.8	992,116	31.5	4109	32.8
Northern Sweden	758,833	25.5	3744	18.0	746,690	23.7	2069	16.5
Marital status								
Married	1,763,061	59.3	13,368	64.3	1,711,978	54.3	7314	58.3
Not married	1,208,719	40.7	7412	35.7	1,439,903	45.7	5227	41.7
Neighborhood deprivation								
Low	641,838	21.6	4581	22.0	688,303	21.8	2896	23.1
Middle	1,584,047	53.3	12,352	59.4	1,730,620	54.9	7395	59.0
High	430,330	14.5	3276	15.8	470,311	14.9	2040	16.3
Unknown	315,565	10.6	571	2.7	262,647	8.3	210	1.7
Diagnosis of diabetes	253,232	8.5	4080	19.6	208,090	6.6	1833	14.6
Diagnosis of COPD	165,770	5.6	2732	13.1	212,661	6.7	2251	17.9
Diagnosis of alcoholism	111,223	3.7	1525	7.3	53,170	1.7	398	3.2
Diagnosis of coronary heart disease	393,288	13.2	7842	37.7	274,604	8.7	4366	34.8
Diagnosis of hypertension	539,167	18.1	9287	44.7	585,632	18.6	6027	48.1
Diagnosis of atrial fibrillation	282,384	9.5	9421	45.3	240,166	7.6	3608	28.8
Diagnosis of stroke	249,927	8.4	3075	14.8	243,457	7.7	1812	14.4
Diagnosis of congestive heart disease	229,979	7.7	15,283	73.5	214,820	6.8	7190	57.3
Diagnosis of amyloidosis	2345	0.1	422	2.0	1832	0.1	168	1.3
Diagnosis of systemic connective tissue disorders	33,014	1.1	461	2.2	78,405	2.5	647	5.2
Diagnosis of sarcoidosis	8889	0.3	149	0.7	7830	0.2	91	0.7
Diagnosis of hemochromatosis	2712	0.1	34	0.2	1795	0.1	25	0.2
Diagnosis of thyroid disorders	30,175	1.0	667	3.2	148,086	4.7	1178	9.4
Diagnosis of chronic rheumatic heart disease	4785	0.2	223	1.1	6524	0.2	168	1.3
Diagnosis of non-rheumatic valvular heart disease	79,755	2.7	2754	13.3	70,819	2.2	1626	13.0
Diagnosis of cancer	556,348	18.7	5212	25.1	561,840	17.8	3342	26.6

**Fig. 1:**
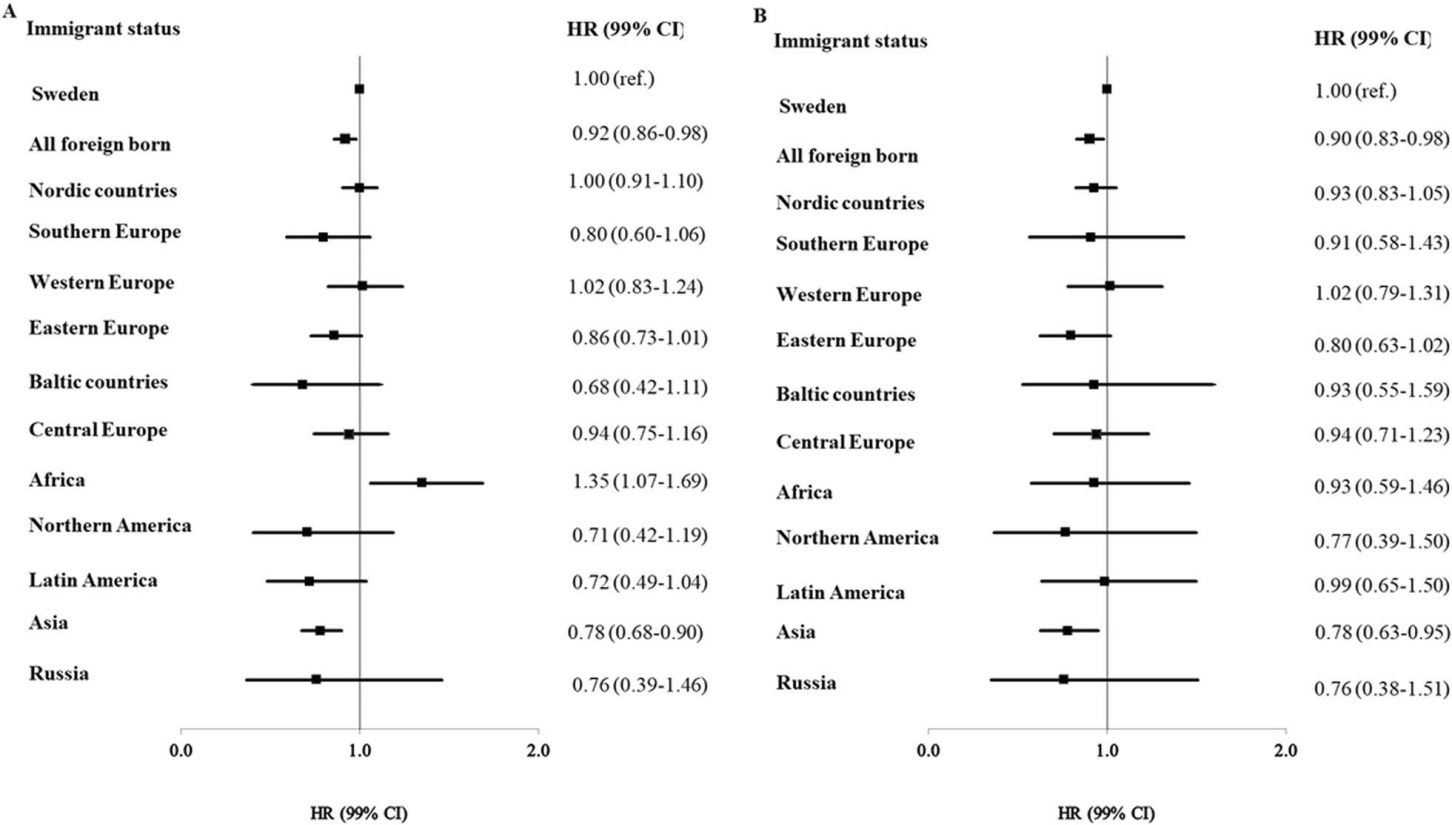
The relative risk of cardiomyopathy in first-generation immigrants vs Swedish-born individuals expressed as hazard ratios (HR) with 99% confidence intervals (99% CI) in men (**A**) and women (**B**)

The relative risk of CMP among foreign-born men and women is shown in Table 2 and Fig. 1. A lower risk of CMP was seen in the fully adjusted models for all foreign-born men, HR 0.92 (99% CI 0.86–0.98), and women, HR 0.90 (99% CI 0.83–0.98). A higher risk was seen in men from Africa, and a lower risk in both men and women from Asia.

**Table 2:** The relative risk of cardiomyopathy in first-generation immigrants vs Swedish-born individuals expressed as hazard ratios (HR) with 99% confidence intervals (99% CI)

	Obs	Model 1			Model 2			Model 3		
		HR	99% CI		HR	99% CI		HR	99% CI	
**Men**										
Sweden	18,214	1			1			1		
All foreign-born	2566	**0.81**	**0.77**	**0.86**	0.99	0.93	1.06	**0.92**	**0.86**	**0.98**
Nordic countries	969	0.82	0.75	0.90	1.07	0.97	1.18	1.00	0.91	1.10
Southern Europe	104	**0.50**	**0.38**	**0.66**	**0.71**	**0.54**	**0.94**	0.80	0.60	1.06
Western Europe	210	0.88	0.72	1.08	1.13	0.93	1.38	1.02	0.83	1.24
Eastern Europe	337	0.91	0.77	1.06	0.95	0.81	1.12	0.86	0.73	1.01
Baltic countries	34	0.73	0.45	1.18	0.79	0.48	1.28	0.68	0.42	1.11
Central Europe	172	1.04	0.84	1.30	1.17	0.94	1.46	0.94	0.75	1.16
Africa	166	**1.25**	**1.00**	**1.56**	**1.45**	**1.16**	**1.82**	**1.35**	**1.07**	**1.69**
Northern America	30	**0.45**	**0.27**	**0.76**	0.69	0.41	1.16	0.71	0.42	1.19
Latin America	59	**0.52**	**0.36**	**0.76**	**0.58**	**0.40**	**0.84**	0.72	0.49	1.04
Asia	458	**0.78**	**0.68**	**0.89**	0.89	0.77	1.02	**0.78**	**0.68**	**0.90**
Russia	19	0.72	0.37	1.38	0.88	0.46	1.69	0.76	0.39	1.46
**Women**										
Sweden	11,042	1			1			1		
All foreign-born	1499	**0.87**	**0.80**	**0.94**	1.00	0.92	1.08	**0.90**	**0.83**	**0.98**
Nordic countries	697	**0.87**	**0.77**	**0.97**	1.03	0.92	1.15	0.93	0.83	1.05
Southern Europe	41	**0.58**	**0.37**	**0.90**	0.86	0.55	1.35	0.91	0.58	1.43
Western Europe	130	1.01	0.79	1.30	1.17	0.91	1.51	1.02	0.79	1.31
Eastern Europe	150	0.88	0.70	1.11	0.89	0.70	1.13	0.80	0.63	1.02
Baltic countries	29	0.95	0.56	1.62	1.01	0.59	1.72	0.93	0.55	1.59
Central Europe	107	1.01	0.76	1.33	1.06	0.80	1.39	0.94	0.71	1.23
Africa	40	0.97	0.62	1.52	1.07	0.68	1.69	0.93	0.59	1.46
Northern America	18	0.58	0.29	1.13	0.83	0.42	1.63	0.77	0.39	1.50
Latin America	48	0.83	0.55	1.26	0.91	0.60	1.38	0.99	0.65	1.50
Asia	221	0.86	0.70	1.04	0.93	0.76	1.14	**0.78**	**0.63**	**0.95**
Russia	17	0.75	0.38	1.50	0.90	0.45	1.79	0.76	0.38	1.51

Significant findings are shown in bold

Model 1: adjusted for age and region of residence in Sweden; model 2: adjusted for age, region of residence in Sweden, educational level, marital status, and neighborhood deprivations; model 3: model 2 + comorbidities

The risk of CMP in individuals ≤ 54 years of age and > 54 years is shown in Table 3. The risk among foreign-born men was lower for both age groups, with fully adjusted HRs of 0.89 (99% CI 0.82–0.97) and of 0.87 (99% CI 0.79–0.96), respectively, and among foreign-born women ≤ 54 years of age, with a HR of 0.84 (99% CI 0.75–0.94). For specific immigrant groups, the risk was higher among men ≤ 54 years of age from Africa, lower among men ≤ 54 years of age from Asia, and men > 54 years from Southern Europe. For foreign-born women, the risk was lower among women ≤ 54 years of age from Nordic countries.

**Table 3:** The relative risk of cardiomyopathy in first-generation immigrants vs. Swedish-born individuals expressed as hazard ratios (HR) with 99% confidence intervals (99% CI) in younger and older individuals

	Obs	≤ 54 years			Obs	> 54 years		
		HR*	99% CI			HR*	99% CI	
**Men**								
Sweden	9455	1			8759	1		
All foreign-born	1592	**0.89**	**0.82**	**0.97**	974	**0.87**	**0.79**	**0.96**
Nordic countries	511	0.92	0.81	1.05	458	0.89	0.77	1.02
Southern Europe	62	0.82	0.57	1.19	**42**	**0.62**	**0.40**	**0.96**
Western Europe	70	0.89	0.63	1.26	140	1.03	0.81	1.32
Eastern Europe	240	0.84	0.70	1.02	97	0.77	0.58	1.04
Baltic countries	5	1.10	0.30	3.93	29	0.73	0.43	1.24
Central Europe	98	0.90	0.67	1.20	74	0.90	0.64	1.26
Africa	144	**1.40**	**1.10**	**1.79**	22	1.31	0.71	2.41
Northern America	12	0.53	0.23	1.20	18	0.99	0.50	1.94
Latin America	52	0.78	0.52	1.16	7	0.48	0.16	1.41
Asia	383	**0.83**	**0.70**	**0.97**	75	0.82	0.59	1.14
Russia	7	0.67	0.23	1.97	12	1.04	0.45	2.36
**Women**								
Sweden	5174	1			5868	1		
All foreign-born	812	**0.84**	**0.75**	**0.94**	687	0.90	0.80	1.02
Nordic countries	303	**0.78**	**0.66**	**0.93**	394	0.88	0.76	1.02
Southern Europe	19	0.75	0.39	1.45	22	0.91	0.49	1.69
Western Europe	33	1.03	0.62	1.69	97	1.02	0.76	1.37
Eastern Europe	113	0.81	0.62	1.07	37	0.69	0.43	1.11
Baltic countries	1	0.27	0.02	4.70	28	1.14	0.67	1.96
Central Europe	64	0.82	0.57	1.18	43	0.99	0.64	1.54
Africa	38	1.17	0.73	1.87	2	1.01	0.13	7.56
Northern America	8	0.99	0.36	2.73	10	0.82	0.33	2.03
Latin America	39	1.07	0.68	1.70	9	1.06	0.41	2.74
Asia	186	0.91	0.72	1.13	35	0.87	0.53	1.41
Russia	7	0.77	0.26	2.26	10	1.10	0.45	2.72

Significant findings are shown in bold

*Fully adjusted

For the different categories of CMPs among men (Table 4), the risk of dilated CMP was higher in men from Nordic countries, more specifically among men from Finland (n = 287, HR 1.42, 99% CI 1.21–1.66), and lower among men from Asia. The risk of hypertrophic CMPs was higher for all foreign-born men, HR 1.22 (99% CI 1.04–1.43), and among men from Africa, and men from Asia; and the risk of other types of CMPs was lower in all foreign-born men, HR 0.89 (99% CI 0.81–0.98), and in men from Asia. For the different categories of CMPs among women (Table 4), the risk of dilated CMP was lower in all foreign-born women, HR 0.77 (99% CI 0.64–0.93), and in women from Asia; and the risk of all other types of CMPs was lower in all foreign-born women, HR 0.89 (99% CI 0.80–0.99), and among women from Asia.

**Table 4:** The relative risk of cardiomyopathy in male first-generation immigrants vs male Swedish-born individuals expressed as hazard ratios (HR) with 99% confidence intervals (99% CI) by type of cardiomyopathy (CMP)

	Obs	Dilated CMP			Obs	Hypertrophic CMP			Obs	Other types		
		HR*	99% CI			HR*	99% CI			HR*	99% CI	
**Men**												
Sweden	6662	1			2325	1			9227	1		
All foreign-born	866	0.90	0.80	1.00	453	**1.22**	**1.04**	**1.43**	1247	**0.89**	**0.81**	**0.98**
Nordic countries	378	**1.26**	**1.09**	**1.45**	104	0.85	0.64	1.14	487	0.97	0.84	1.10
Southern Europe	34	0.97	0.60	1.55	20	1.11	0.58	2.11	50	0.75	0.50	1.12
Western Europe	71	1.07	0.77	1.48	30	1.08	0.64	1.84	109	1.01	0.76	1.32
Eastern Europe	111	0.80	0.60	1.06	55	1.13	0.76	1.67	171	0.91	0.72	1.13
Baltic countries	14	0.63	0.27	1.47	1	0.17	0.01	2.92	19	0.70	0.36	1.34
Central Europe	55	0.85	0.58	1.26	35	1.55	0.95	2.52	82	0.86	0.63	1.19
Africa	44	0.86	0.48	1.53	45	**2.81**	**1.81**	**4.38**	77	1.33	0.95	1.85
Northern America	10	0.78	0.30	2.00	4	0.68	0.16	2.84	16	0.74	0.36	1.50
Latin America	14	0.71	0.32	1.56	12	1.00	0.44	2.29	33	0.83	0.50	1.37
Asia	128	**0.67**	**0.49**	**0.92**	141	**1.83**	**1.40**	**2.40**	189	**0.68**	**0.55**	**0.85**
Russia	6	0.58	0.14	2.50	4	1.25	0.30	5.21	9	0.70	0.27	1.82
**Women**												
Sweden	2619	1			2013	1			6410	1		
All foreign-born	306	**0.77**	**0.64**	**0.93**	322	1.14	0.95	1.36	871	**0.89**	**0.80**	**0.99**
Nordic countries	151	1.03	0.83	1.29	123	0.90	0.69	1.17	423	0.95	0.82	1.10
Southern Europe	8	0.94	0.39	2.24	13	1.73	0.78	3.85	20	0.75	0.39	1.42
Western Europe	15	0.63	0.31	1.28	31	1.26	0.75	2.11	84	1.12	0.82	1.53
Eastern Europe	40	0.77	0.47	1.25	37	1.32	0.81	2.13	73	0.68	0.49	0.96
Baltic countries	4	0.49	0.09	2.64	8	1.25	0.46	3.45	17	0.94	0.47	1.87
Central Europe	18	0.68	0.33	1.41	28	1.47	0.85	2.54	61	0.90	0.62	1.30
Africa	12	0.71	0.25	2.03	**7**	1.35	0.45	4.00	21	0.87	0.47	1.63
Northern America	1	0.47	0.05	4.13	5	1.28	0.36	4.61	12	0.86	0.38	1.96
Latin America	8	0.72	0.26	2.02	12	1.67	0.73	3.83	28	0.97	0.57	1.68
Asia	47	**0.55**	**0.33**	**0.90**	51	1.37	0.90	2.09	123	**0.76**	**0.58**	**0.99**
Russia	2	0.43	0.03	5.44	7	1.93	0.65	5.68	8	0.61	0.22	1.66

Significant findings are shown in bold

*Fully adjusted

In the second-generation immigrant study, a total of 4,587,764 individuals were included, 2,345,774 men and 2,241,990 women, including 275,105 men and 257,922 women with foreign-born parents (Additional file 1: Table S5). In total, 26,559 cases of CMP were registered, 17,620 among men and 8939 among women, including 1500 immigrant men and 730 immigrant women. No statistically significant results were found (Additional file 1: Tables S2–S4), where the fully models adjusted showed HRs (99% CI) for all CMPs among men 0.96 (0.89–1.04) and women 0.97 (0.86–1.08); in men and women combined for ≤ 54 years of age 0.97 (0.90–1.04) and for > 54 years 0.98 (0.83–1.16); and in men and women combined for dilated CMPs HR 0.97 (0.86–1.10), hypertrophic CMPs 0.94 (0.79–1.12), and all other types 1.00 (0.91–1.09).

In the sensitivity analyses, we analyzed the effect of cancer specifically on the risk of cardiomyopathy (Additional file 1: Tables S6 and S7). In the first-generation study, the HRs adjusted for age and all cancers were lower for foreign-born men in general, HR 0.79 (99% CI 0.75–0.84), and among men from Southern Europe, Eastern Europe, Northern America, Latin America, and Asia; and for foreign-born women in general, HR 0.71 (995 CI 0.75–0.87), and among women from Eastern Europe, Africa, Latin America, and Asia (Additional file 1: Table S6). In the second-generation study, the corresponding HRs were lower for all men with foreign-born parents, HR 0.73 (99% CI 0.68–0.79); for men with parents from all European regions except Baltic countries; for men with parents from Latin America and Asia; for all women with foreign-born parents, HR 0.73 (99% CI 0.65–0.82), and women with parents from the Nordic countries, Eastern Europe, and Asia (Additional file 1: Table S7).

## Discussion

Our main findings were that when including all CMPs, the risk was, in general, lower in both foreign-born men and women. A lower risk was found among men and women from Asia, while a higher risk was seen among men from Africa. Regarding age patterns, a lower risk of overall CMP was found among men both for those ≤ 54 years of age, and > 54 years. However, for women it was statistically significant only in the younger age group. There were no significant differences between second-generation immigrants compared to native Swedes.

Studies on other diseases in immigrants in Sweden have found a higher risk in many immigrant groups as regards CHD [17], a higher risk of CHF [18], and also of AF among individuals younger than 45 years of age [19], especially in immigrants from some Middle Eastern countries. Furthermore, the risk of type 2 diabetes is higher in immigrants, particularly those from the Middle East region [17].

The lower risk in general for CMPs could be due to the so-called “healthy migrant effect” [20], i.e. that migrating individuals are healthier than their compatriots in the country of origin and that, in this case, individuals with CMPs, to a higher extent, stayed in their home countries. Thus, groups with increased risks could be more important to identify. Hypertrophic CMPs remain the most common inherited heart disease thus making them of special interest to study among immigrants [5], as there are genetic differences between immigrant groups. The risk was higher in men from African and Asian countries, while not among women, perhaps owing to too low case numbers. Dilated CMPs could also be of familial type [3], but we only found an increased risk among men from the Nordic countries, which was driven by an increased risk among men from Finland, perhaps because of differences in risk factors between population groups.

The correlation of CMPs to CHF is of clinical importance. We found a presence of CHF in the first-generation study of 74% among men and 57% among women, with corresponding rates in the second-generation study of 75% and 56%, respectively. In an earlier Swedish immigrant study conducted among individuals aged 45 years and older, the population attributable fraction (PAF) of CMPs for incident CHF was 4.6% for Swedish-born men and 5.7% for foreign-born men, and it was 2.1% for Swedish-born women and 2.4% for foreign-born women [18].

The correlation between CMPs and atrial fibrillation (AF) is also of clinical importance. We found that the rate of AF in the first-generation study among men was 45% and among women the figure was 29%, and that the corresponding rates in the second-generation study were 43% for men and 23% for women. The risk of AF among most first- and second-generation men and women in Sweden has been found to be lower both in those aged 45 years of age and older [21] and in those < 45 years of age [19]. For the younger individuals, i.e. younger than 45 years of age, the PAFs for CMPs regarding incident AF were 2.9% for Swedish-born men and 4.1% for foreign-born men, 3.1% for Swedish-born women and 1.4% for foreign-born women [19].

Not surprisingly, the rates of CHD, hypertension and, to some extent, also of diabetes, COPD, stroke, and non-rheumatic valvular heart disease, were also high, as was the rate of cancer. When only adjusted for age and cancers, the HRs were lower in general, and also for many of the studied groups.

There are certain limitations of this study. We used three large groups, thus not being able to follow the proposal from the ESC [2]. Dilated and hypertrophic CMPs are the most important and prevalent types of CMPs. However, we included them separately and the other types are less prevalent, hence why we merged them into one group for practical and analytical reasons in order to obtain more statistical power. We used diagnoses from the NPR, based on clinical diagnoses from patient records, with no possibility to check diagnostic criteria. However, the diagnoses were obtained from hospital diagnoses, where most patients are seen by specialists at least once. As most individuals with CMPs are examined in hospitals, the coverage of patients with these diagnoses could be expected to be high, even if some cases may be undiagnosed. We did not include ischemic cardiomyopathy (I25.5), and we cannot rule out that some diagnoses are misclassified, but we have no possibility to check for this in the nationwide data that we used. In total, 7191 men and 1943 women were registered with a diagnosis of ischemic cardiomyopathy, and of these 1042 men (14.5%) and 195 women (10.0%) also were registered with another CMP diagnosis. In addition, diagnoses from primary care were not included and most patients with comorbidities, such as hypertension and diabetes, are treated in primary care.

Our study also has several strengths. In Sweden, personal identity numbers allow linkage between different national Swedish registers [22] thus enabling adjustments for many potential confounding factors. Furthermore, many Swedish registers have been shown to have good quality [23, 24].

In conclusion, we found a generally lower risk of CMPs among foreign-born men and women in Sweden, possibly owing to the “healthy migrant effect”, but a higher risk in some specific groups, i.e. for dilated CMP among men from Finland, and for hypertrophic CMP among men from Africa and Asian countries. Hereditary forms of CMPs seem to be of little importance on a population level, as there were no significant findings when we studied second-generation immigrants.

## Supplementary Information

**Additional file 1**. Supplementary tables.

## Data Availability

The datasets generated and/or analyzed during the current study are not publicly available due to restraints in the ethical approvals but we are willing to collaborate upon request to the corresponding author.
