# Supplementary material for: Cardiomyopathy among first- and second-generation immigrants in Sweden: a nationwide total population study
Source: BMC Cardiovasc Disord. 2022 Dec 6;22:524. doi: 10.1186/s12872-022-02968-0 (PMC9724305; doi:10.1186/s12872-022-02968-0)
Supplement: Supplementary file 1 — Additional file 1. Supplementary tables. [file 12872_2022_2968_MOESM1_ESM.docx]

| **Supplementary Table 1a. The study population in the first-generation study and number of cardiomyopathy cases (CMPs) in men** | | | | | | | | | | | | |  |  |  |  |
| --- | --- | --- | --- | --- | --- | --- | --- | --- | --- | --- | --- | --- | --- | --- | --- | --- |
|  | Swedish-born | | | | | | | |  | | Foreign-born | | | | | |
|  | Population | | |  | | CMPs | | |  | | Population | | |  | CMPs | |
|  | No. | % |  | | No | | % |  | | No. | | % | |  | No | % |
| Total population | 2447554 |  |  | | 18214 | |  |  | | 524226 | |  | |  | 2566 |  |
| Age (years) |  |  |  | |  | |  |  | |  | |  | |  |  |  |
| 18-39 | 908378 | 37.1 |  | | 2821 | | 15.5 |  | | 266959 | | 50.9 | |  | 601 | 23.4 |
| 40-49 | 418802 | 17.1 |  | | 3668 | | 20.1 |  | | 95867 | | 18.3 | |  | 625 | 24.4 |
| 50-59 | 448652 | 18.3 |  | | 5601 | | 30.8 |  | | 82998 | | 15.8 | |  | 732 | 28.5 |
| 60+ | 671722 | 27.4 |  | | 6124 | | 33.6 |  | | 78402 | | 15.0 | |  | 608 | 23.7 |
| Educational level |  |  |  | |  | |  |  | |  | |  | |  |  |  |
| ≤ 9 | 808765 | 33.0 |  | | 6878 | | 37.8 |  | | 315722 | | 60.2 | |  | 1135 | 44.2 |
| 10-12 | 1063694 | 43.5 |  | | 7691 | | 42.2 |  | | 124852 | | 23.8 | |  | 930 | 36.2 |
| > 12 | 575095 | 23.5 |  | | 3645 | | 20.0 |  | | 83652 | | 16.0 | |  | 501 | 19.5 |
| Region of residence |  |  |  | |  | |  |  | |  | |  | |  |  |  |
| Large cities | 1106272 | 45.2 |  | | 8964 | | 49.2 |  | | 191470 | | 36.5 | |  | 1467 | 57.2 |
| Southern Sweden | 829241 | 33.9 |  | | 6013 | | 33.0 |  | | 85964 | | 16.4 | |  | 592 | 23.1 |
| Northern Sweden | 512041 | 20.9 |  | | 3237 | | 17.8 |  | | 246792 | | 47.1 | |  | 507 | 19.8 |
| Marital status |  |  |  | |  | |  |  | |  | |  | |  |  |  |
| Married | 1357831 | 55.5 |  | | 11615 | | 63.8 |  | | 405230 | | 77.3 | |  | 1753 | 68.3 |
| Not married | 1089723 | 44.5 |  | | 6599 | | 36.2 |  | | 118996 | | 22.7 | |  | 813 | 31.7 |
| Neighborhood deprivation |  |  |  | |  | |  |  | |  | |  | |  |  |  |
| Low | 591052 | 24.1 |  | | 4196 | | 23.0 |  | | 50786 | | 9.7 | |  | 385 | 15.0 |
| Middle | 1447182 | 59.1 |  | | 11188 | | 61.4 |  | | 136865 | | 26.1 | |  | 1164 | 45.4 |
| High | 316659 | 12.9 |  | | 2579 | | 14.2 |  | | 113671 | | 21.7 | |  | 697 | 27.2 |
| Unknown | 92661 | 3.8 |  | | 251 | | 1.4 |  | | 222904 | | 42.5 | |  | 320 | 12.5 |
| Diagnosis of diabetes | 212310 | 8.7 |  | | 3417 | | 18.8 |  | | 40922 | | 7.8 | |  | 663 | 25.8 |
| Diagnosis of COPD | 142578 | 5.8 |  | | 2321 | | 12.7 |  | | 23192 | | 4.4 | |  | 411 | 16.0 |
| Diagnosis of alcoholism | 96153 | 3.9 |  | | 1327 | | 7.3 |  | | 15070 | | 2.9 | |  | 198 | 7.7 |
| Diagnosis of coronary heart disease | 342813 | 14.0 |  | | 6720 | | 36.9 |  | | 50475 | | 9.6 | |  | 1122 | 43.7 |
| Diagnosis of hypertension | 473394 | 19.3 |  | | 8106 | | 44.5 |  | | 65773 | | 12.5 | |  | 1181 | 46.0 |
| Diagnosis of atrial fibrillation | 257205 | 10.5 |  | | 8409 | | 46.2 |  | | 25179 | | 4.8 | |  | 1012 | 39.4 |
| Diagnosis of stroke | 224217 | 9.2 |  | | 2699 | | 14.8 |  | | 25710 | | 4.9 | |  | 376 | 14.7 |
| Diagnosis of congestive heart disease | 206960 | 8.5 |  | | 13474 | | 74.0 |  | | 23019 | | 4.4 | |  | 1809 | 70.5 |
| Diagnosis of amyloidosis | 1991 | 0.1 |  | | 388 | | 2.1 |  | | 354 | | 0.1 | |  | 34 | 1.3 |
| Diagnosis of systemic connective tissue disorders | 29933 | 1.2 |  | | 415 | | 2.3 |  | | 3081 | | 0.6 | |  | 46 | 1.8 |
| Diagnosis of sarcoidosis | 8054 | 0.3 |  | | 139 | | 0.8 |  | | 835 | | 0.2 | |  | 10 | 0.4 |
| Diagnosis of hemochromatosis | 2488 | 0.1 |  | | 32 | | 0.2 |  | | 224 | | 0.0 | |  | 2 | 0.1 |
| Diagnosis of thyroid disorders | 25332 | 1.0 |  | | 577 | | 3.2 |  | | 4843 | | 0.9 | |  | 90 | 3.5 |
| Diagnosis of chronic rheumatic heart disease | 4010 | 0.2 |  | | 191 | | 1.0 |  | | 775 | | 0.1 | |  | 32 | 1.2 |
| Diagnosis of non-rheumatic valvular heart disease | 71701 | 2.9 |  | | 2438 | | 13.4 |  | | 8054 | | 1.5 | |  | 316 | 12.3 |
| Diagnosis of cancer | 503122 | 20.6 |  | | 4690 | | 25.7 |  | | 53226 | | 10.2 | |  | 522 | 20.3 |

| **Supplementary Table 1b. The study population in first-generation study and number of cardiomyopathy cases (CMPs) in women** | | | | | | | | | | |  | |  | |
| --- | --- | --- | --- | --- | --- | --- | --- | --- | --- | --- | --- | --- | --- | --- |
|  | Swedish-born | | | | |  | Foreign-born | | | | | | |  |
|  | Population | |  | CMPs | |  | Population | |  | CMPs | | | |  |
|  | No. | % |  | No | % |  | No. | % |  | No | | % | |  |
| Total population | 2641121 |  |  | 11042 |  |  | 510760 |  |  | 1499 | |  | |  |
| Age (years) |  |  |  |  |  |  |  |  |  |  | |  | |  |
| 18-39 | 904824 | 34.3 |  | 1527 | 13.8 |  | 257043 | 50.3 |  | 299 | | 19.9 | |  |
| 40-49 | 433225 | 16.4 |  | 1944 | 17.6 |  | 94269 | 18.5 |  | 315 | | 21.0 | |  |
| 50-59 | 462868 | 17.5 |  | 3366 | 30.5 |  | 71990 | 14.1 |  | 396 | | 26.4 | |  |
| 60+ | 840204 | 31.8 |  | 4205 | 38.1 |  | 87458 | 17.1 |  | 489 | | 32.6 | |  |
| Educational level |  |  |  |  |  |  |  |  |  |  | |  | |  |
| ≤ 9 | 867117 | 32.8 |  | 4170 | 37.8 |  | 297027 | 58.2 |  | 715 | | 47.7 | |  |
| 10-12 | 1119291 | 42.4 |  | 4615 | 41.8 |  | 125175 | 24.5 |  | 502 | | 33.5 | |  |
| > 12 | 654713 | 24.8 |  | 2257 | 20.4 |  | 88558 | 17.3 |  | 282 | | 18.8 | |  |
| Region of residence |  |  |  |  |  |  |  |  |  |  | |  | |  |
| Large cities | 1209616 | 45.8 |  | 5463 | 49.5 |  | 203459 | 39.8 |  | 900 | | 60.0 | |  |
| Southern Sweden | 900508 | 34.1 |  | 3754 | 34.0 |  | 91608 | 17.9 |  | 355 | | 23.7 | |  |
| Northern Sweden | 530997 | 20.1 |  | 1825 | 16.5 |  | 215693 | 42.2 |  | 244 | | 16.3 | |  |
| Marital status |  |  |  |  |  |  |  |  |  |  | |  | |  |
| Married | 1334083 | 50.5 |  | 6400 | 58.0 |  | 377895 | 74.0 |  | 914 | | 61.0 | |  |
| Not married | 1307038 | 49.5 |  | 4642 | 42.0 |  | 132865 | 26.0 |  | 585 | | 39.0 | |  |
| Neighborhood deprivation |  |  |  |  |  |  |  |  |  |  | |  | |  |
| Low | 627986 | 23.8 |  | 2615 | 23.7 |  | 60317 | 11.8 |  | 281 | | 18.7 | |  |
| Middle | 1575928 | 59.7 |  | 6715 | 60.8 |  | 154692 | 30.3 |  | 680 | | 45.4 | |  |
| High | 358651 | 13.6 |  | 1629 | 14.8 |  | 111660 | 21.9 |  | 411 | | 27.4 | |  |
| Unknown | 78556 | 3.0 |  | 83 | 0.8 |  | 184091 | 36.0 |  | 127 | | 8.5 | |  |
| Diagnosis of diabetes | 178164 | 6.7 |  | 1561 | 14.1 |  | 29926 | 5.9 |  | 272 | | 18.1 | |  |
| Diagnosis of COPD | 184371 | 7.0 |  | 1986 | 18.0 |  | 28290 | 5.5 |  | 265 | | 17.7 | |  |
| Diagnosis of alcoholism | 45928 | 1.7 |  | 348 | 3.2 |  | 7242 | 1.4 |  | 50 | | 3.3 | |  |
| Diagnosis of coronary heart disease | 242975 | 9.2 |  | 3763 | 34.1 |  | 31629 | 6.2 |  | 603 | | 40.2 | |  |
| Diagnosis of hypertension | 514890 | 19.5 |  | 5248 | 47.5 |  | 70742 | 13.9 |  | 779 | | 52.0 | |  |
| Diagnosis of atrial fibrillation | 217432 | 8.2 |  | 3181 | 28.8 |  | 22734 | 4.5 |  | 427 | | 28.5 | |  |
| Diagnosis of stroke | 219679 | 8.3 |  | 1578 | 14.3 |  | 23778 | 4.7 |  | 234 | | 15.6 | |  |
| Diagnosis of congestive heart disease | 195077 | 7.4 |  | 6379 | 57.8 |  | 19743 | 3.9 |  | 811 | | 54.1 | |  |
| Diagnosis of amyloidosis | 1547 | 0.1 |  | 152 | 1.4 |  | 285 | 0.1 |  | 16 | | 1.1 | |  |
| Diagnosis of systemic connective tissue disorders | 69975 | 2.6 |  | 578 | 5.2 |  | 8430 | 1.7 |  | 69 | | 4.6 | |  |
| Diagnosis of sarcoidosis | 6808 | 0.3 |  | 83 | 0.8 |  | 1022 | 0.2 |  | 8 | | 0.5 | |  |
| Diagnosis of hemochromatosis | 1672 | 0.1 |  | 24 | 0.2 |  | 123 | 0.0 |  | 1 | | 0.1 | |  |
| Diagnosis of thyroid disorders | 126211 | 4.8 |  | 1029 | 9.3 |  | 21875 | 4.3 |  | 149 | | 9.9 | |  |
| Diagnosis of chronic rheumatic heart disease | 5397 | 0.2 |  | 130 | 1.2 |  | 1127 | 0.2 |  | 38 | | 2.5 | |  |
| Diagnosis of non-rheumatic valvular heart diseases | 63332 | 2.4 |  | 1433 | 13.0 |  | 7487 | 1.5 |  | 193 | | 12.9 | |  |
| Diagnosis of cancer | 506789 | 19.2 |  | 3000 | 27.2 |  | 55051 | 10.8 |  | 342 | | 22.8 | |  |

| **Supplementary Table 2. The relative risk of cardiomyopathy in male and female second-generation immigrants vs native Swedes expressed as hazard ratios (HR) with 99% confidence intervals (99% CI)** | | | | | | | | | | | | |
| --- | --- | --- | --- | --- | --- | --- | --- | --- | --- | --- | --- | --- |
|  |  | Model 1 | | |  | Model 2 | | |  | Model 3 | | |
|  | Obs. | HR | 99% CI | |  | HR | 99% CI | |  | HR | 99% CI | |
| **Men** |  |  |  |  |  |  |  |  |  |  |  |  |
| Sweden | 16120 | 1 |  |  |  | 1 |  |  |  | 1 |  |  |
| All with foreign-born parents | 1500 | 1.01 | 0.93 | 1.09 |  | 0.99 | 0.91 | 1.07 |  | 0.96 | 0.89 | 1.04 |
| Nordic countries | 966 | 1.10 | 1.00 | 1.21 |  | 1.07 | 0.97 | 1.18 |  | 1.03 | 0.93 | 1.13 |
| Southern Europe | 43 | 0.94 | 0.61 | 1.45 |  | 0.89 | 0.58 | 1.38 |  | 0.86 | 0.56 | 1.33 |
| Western Europe | 149 | 0.86 | 0.67 | 1.08 |  | 0.87 | 0.68 | 1.10 |  | 0.93 | 0.73 | 1.18 |
| Eastern Europe | 68 | 1.03 | 0.72 | 1.45 |  | 0.95 | 0.67 | 1.34 |  | 0.84 | 0.59 | 1.19 |
| Baltic countries | 62 | 0.85 | 0.59 | 1.21 |  | 0.86 | 0.60 | 1.23 |  | 0.91 | 0.64 | 1.31 |
| Central Europe | 57 | 0.80 | 0.54 | 1.16 |  | 0.79 | 0.54 | 1.15 |  | 0.77 | 0.52 | 1.12 |
| Africa | 9 | 0.93 | 0.36 | 2.42 |  | 0.87 | 0.34 | 2.25 |  | 0.76 | 0.29 | 1.95 |
| Northern America | 59 | 1.00 | 0.69 | 1.44 |  | 1.02 | 0.70 | 1.47 |  | 1.02 | 0.70 | 1.48 |
| Latin America | 9 | 0.57 | 0.22 | 1.46 |  | 0.51 | 0.20 | 1.31 |  | 0.53 | 0.20 | 1.36 |
| Asia | 55 | 0.86 | 0.58 | 1.27 |  | 0.78 | 0.53 | 1.15 |  | 0.73 | 0.49 | 1.07 |
| Russia | 20 | 0.87 | 0.46 | 1.64 |  | 0.87 | 0.46 | 1.64 |  | 0.83 | 0.44 | 1.57 |
|  |  |  |  |  |  |  |  |  |  |  |  |  |
| **Women** |  |  |  |  |  |  |  |  |  |  |  |  |
| Sweden | 8209 | 1 |  |  |  | 1 |  |  |  | 1 |  |  |
| All with foreign-born parents | 730 | 1.02 | 0.91 | 1.14 |  | 1.00 | 0.89 | 1.12 |  | 0.97 | 0.86 | 1.08 |
| Nordic countries | 453 | 1.05 | 0.92 | 1.21 |  | 1.03 | 0.90 | 1.19 |  | 1.00 | 0.87 | 1.15 |
| Southern Europe | 23 | 1.15 | 0.63 | 2.08 |  | 1.08 | 0.60 | 1.96 |  | 1.10 | 0.61 | 2.00 |
| Western Europe | 80 | 0.99 | 0.72 | 1.37 |  | 0.99 | 0.72 | 1.36 |  | 0.97 | 0.71 | 1.34 |
| Eastern Europe | 21 | 0.72 | 0.38 | 1.34 |  | 0.66 | 0.35 | 1.23 |  | 0.55 | 0.30 | 1.03 |
| Baltic countries | 45 | 1.20 | 0.78 | 1.83 |  | 1.19 | 0.78 | 1.83 |  | 1.18 | 0.77 | 1.81 |
| Central Europe | 36 | 1.04 | 0.65 | 1.68 |  | 1.02 | 0.63 | 1.64 |  | 1.04 | 0.64 | 1.67 |
| Africa | 7 | 1.58 | 0.54 | 4.65 |  | 1.51 | 0.51 | 4.43 |  | 1.20 | 0.41 | 3.54 |
| Northern America | 31 | 1.00 | 0.60 | 1.67 |  | 1.02 | 0.61 | 1.70 |  | 1.18 | 0.71 | 1.97 |
| Latin America | 4 | 0.56 | 0.14 | 2.33 |  | 0.51 | 0.12 | 2.11 |  | 0.45 | 0.11 | 1.86 |
| Asia | 21 | 0.78 | 0.42 | 1.46 |  | 0.71 | 0.38 | 1.32 |  | 0.60 | 0.32 | 1.12 |
| Russia | 8 | 0.66 | 0.24 | 1.81 |  | 0.66 | 0.24 | 1.80 |  | 0.58 | 0.21 | 1.60 |
| Model 1: adjusted for age and region of residence in Sweden; model 2: adjusted for age, region of residence in Sweden, educational level, marital status, and neighborhood deprivations; model 3: model 2 + comorbidities. | | | | | | | | | | | | |

| **Supplementary Table 3. The relative risk of cardiomyopathy (CMP) in second-generation immigrants vs native Swedes expressed as hazard ratios (HR) with 99% confidence intervals (99% CI)** | | | | | | | | | | | | | | | |
| --- | --- | --- | --- | --- | --- | --- | --- | --- | --- | --- | --- | --- | --- | --- | --- |
|  |  | Dilated CMP | | |  |  | Hypertrophic CMP | | |  |  | Other types | | |  |
|  | Obs. | HR | 99% CI | |  | Obs. | HR | 99% CI | |  | Obs. | HR | 99% CI | |  |
| Sweden | 8508 | 1 |  |  |  | 3294 | 1 |  |  |  | 12527 | 1 |  |  |  |
| All with foreign-born parents | 761 | 0.97 | 0.86 | 1.10 |  | 298 | 0.94 | 0.79 | 1.12 |  | 1171 | 1.00 | 0.91 | 1.09 |  |
| Nordic countries | 525 | 1.05 | 0.91 | 1.22 |  | 164 | 0.89 | 0.70 | 1.12 |  | 730 | 1.04 | 0.93 | 1.16 |  |
| Southern Europe | 26 | 1.01 | 0.45 | 2.26 |  | 12 | 1.22 | 0.53 | 2.78 |  | 28 | 0.78 | 0.46 | 1.34 |  |
| Western Europe | 67 | 0.91 | 0.62 | 1.34 |  | 33 | 0.94 | 0.57 | 1.54 |  | 129 | 1.03 | 0.80 | 1.33 |  |
| Eastern Europe | 32 | 0.89 | 0.43 | 1.85 |  | 11 | 0.69 | 0.29 | 1.64 |  | 46 | 0.79 | 0.51 | 1.20 |  |
| Baltic countries | 29 | 0.89 | 0.53 | 1.48 |  | 13 | 0.86 | 0.39 | 1.89 |  | 65 | 1.17 | 0.82 | 1.67 |  |
| Central Europe | 19 | 0.79 | 0.41 | 1.52 |  | 20 | 1.26 | 0.66 | 2.39 |  | 54 | 0.95 | 0.65 | 1.41 |  |
| Africa | 6 | 0.97 | 0.15 | 6.26 |  | 1 | 0.43 | 0.02 | 7.41 |  | 9 | 1.01 | 0.39 | 2.63 |  |
| Northern America | 31 | 1.08 | 0.66 | 1.76 |  | 15 | 1.27 | 0.60 | 2.65 |  | 44 | 1.00 | 0.65 | 1.54 |  |
| Latin America | 3 | 0.77 | 0.07 | 8.03 |  | 3 | 0.80 | 0.15 | 4.18 |  | 7 | 0.53 | 0.18 | 1.55 |  |
| Asia | 15 | 0.78 | 0.27 | 2.23 |  | 21 | 1.38 | 0.73 | 2.60 |  | 40 | 0.71 | 0.45 | 1.13 |  |
| Russia | 7 | 0.68 | 0.27 | 1.74 |  | 4 | 0.79 | 0.19 | 3.28 |  | 17 | 0.84 | 0.42 | 1.68 |  |
| Model 1: adjusted for age and region of residence in Sweden; model 2: adjusted for age, region of residence in Sweden, educational level, marital status, and neighborhood deprivations; model 3: model 2 + comorbidities. | | | | | | | | | | | | | | | |

| **Supplementary Table 4. The relative risk of cardiomyopathy in second-generation immigrants vs native Swedes expressed as hazard ratios (HR) with 99% confidence intervals (99% CI)** | | | | | | | | | | | | | | |
| --- | --- | --- | --- | --- | --- | --- | --- | --- | --- | --- | --- | --- | --- | --- |
|  |  | ≤ 54 years | | | |  | | |  | | > 54 years | | | |
|  | Obs. | HR* | 99% CI | | | |  | | | Obs. | | HR* | 99% CI | |
| Sweden | 16210 | 1 |  |  |  | | | 8119 | | | | 1 |  |  |
| All with foreign-born parents | 1931 | 0.97 | 0.90 | 1.04 |  | | | 299 | | | | 0.98 | 0.83 | 1.16 |
| Nordic countries | 1252 | 1.00 | 0.92 | 1.09 |  | | | 167 | | | | 0.99 | 0.79 | 1.24 |
| Southern Europe | 63 | 1.01 | 0.71 | 1.45 |  | | | 3 | | | | 0.77 | 0.15 | 3.98 |
| Western Europe | 186 | 0.95 | 0.77 | 1.17 |  | | | 43 | | | | 1.03 | 0.67 | 1.59 |
| Eastern Europe | 87 | 0.83 | 0.61 | 1.13 |  | | | 2 | | | | 0.95 | 0.13 | 7.16 |
| Baltic countries | 78 | 1.01 | 0.73 | 1.40 |  | | | 29 | | | | 0.92 | 0.54 | 1.57 |
| Central Europe | 82 | 0.83 | 0.60 | 1.13 |  | | | 11 | | | | 1.18 | 0.50 | 2.79 |
| Africa | 16 | 1.12 | 0.55 | 2.28 |  | | |  | | | |  |  |  |
| Northern America | 54 | 1.06 | 0.72 | 1.56 |  | | | 36 | | | | 1.10 | 0.68 | 1.77 |
| Latin America | 13 | 0.61 | 0.28 | 1.34 |  | | |  | | | |  |  |  |
| Asia | 74 | 0.81 | 0.58 | 1.14 |  | | | 2 | | | | 1.25 | 0.17 | 9.40 |
| Russia | 23 | 0.85 | 0.47 | 1.53 |  | | | 5 | | | | 0.41 | 0.11 | 1.46 |
| *. Full adjusted. | | | | | | | | | | | | | | |

| **Supplementary Table 5. The population in the second-generation study and the number of cardiomyopathy (CMP) cases categorized by sex** | | | | | | | | | | | |
| --- | --- | --- | --- | --- | --- | --- | --- | --- | --- | --- | --- |
|  | Men | | | | |  | Women | | | | |
|  | Population | |  | CMPs | |  | Population | |  | CMPs | |
|  | Number | % |  | Number | % |  | Number | % |  | Number | % |
| Total population | 2345774 |  |  | 17620 |  |  | 2241990 |  |  | 8939 |  |
| Immigrant status |  |  |  |  |  |  |  |  |  |  |  |
| Swedish | 2070669 | 88.3 |  | 16120 | 91.5 |  | 1984068 | 88.5 |  | 8209 | 91.8 |
| Foreign born | 275105 | 11.7 |  | 1500 | 8.5 |  | 257922 | 11.5 |  | 730 | 8.2 |
| Age (years) |  |  |  |  |  |  |  |  |  |  |  |
| 18-39 | 1173236 | 50.0 |  | 4115 | 23.4 |  | 1106115 | 49.3 |  | 1902 | 21.3 |
| 40-49 | 506286 | 21.6 |  | 4751 | 27.0 |  | 483228 | 21.6 |  | 2166 | 24.2 |
| 50-59 | 486671 | 20.7 |  | 6199 | 35.2 |  | 470216 | 21.0 |  | 3372 | 37.7 |
| 60+ | 179581 | 7.7 |  | 2555 | 14.5 |  | 182431 | 8.1 |  | 1499 | 16.8 |
| Educational level |  |  |  |  |  |  |  |  |  |  |  |
| ≤ 9 | 617133 | 26.3 |  | 6068 | 34.4 |  | 507667 | 22.6 |  | 2680 | 30.0 |
| 10-12 | 1103926 | 47.1 |  | 7976 | 45.3 |  | 1065165 | 47.5 |  | 4139 | 46.3 |
| > 12 | 624715 | 26.6 |  | 3576 | 20.3 |  | 669158 | 29.8 |  | 2120 | 23.7 |
| Region of residence |  |  |  |  |  |  |  |  |  |  |  |
| Large cities | 1110184 | 47.3 |  | 8671 | 49.2 |  | 1080007 | 48.2 |  | 4428 | 49.5 |
| Southern Sweden | 806277 | 34.4 |  | 5866 | 33.3 |  | 763856 | 34.1 |  | 3063 | 34.3 |
| Northern Sweden | 429313 | 18.3 |  | 3083 | 17.5 |  | 398127 | 17.8 |  | 1448 | 16.2 |
| Marital status |  |  |  |  |  |  |  |  |  |  |  |
| Married | 966316 | 41.2 |  | 8720 | 49.5 |  | 1026055 | 45.8 |  | 4947 | 55.3 |
| Not married | 1379458 | 58.8 |  | 8900 | 50.5 |  | 1215935 | 54.2 |  | 3992 | 44.7 |
| Neighborhood deprivation |  |  |  |  |  |  |  |  |  |  |  |
| Low | 578042 | 24.6 |  | 3885 | 22.0 |  | 577608 | 25.8 |  | 2171 | 24.3 |
| Middle | 1420456 | 60.6 |  | 10860 | 61.6 |  | 1348875 | 60.2 |  | 5439 | 60.8 |
| High | 339903 | 14.5 |  | 2842 | 16.1 |  | 310390 | 13.8 |  | 1318 | 14.7 |
| Unknown | 7373 | 0.3 |  | 33 | 0.2 |  | 5117 | 0.2 |  | 11 | 0.1 |
| Diagnosis of diabetes | 177020 | 7.5 |  | 3472 | 19.7 |  | 111688 | 5.0 |  | 1244 | 13.9 |
| Diagnosis of COPD | 107511 | 4.6 |  | 2107 | 12.0 |  | 140548 | 6.3 |  | 1593 | 17.8 |
| Diagnosis of alcoholism | 117775 | 5.0 |  | 1668 | 9.5 |  | 51538 | 2.3 |  | 373 | 4.2 |
| Diagnosis of coronary heart disease | 211166 | 9.0 |  | 5666 | 32.2 |  | 99340 | 4.4 |  | 2613 | 29.2 |
| Diagnosis of hypertension | 398704 | 17.0 |  | 8075 | 45.8 |  | 330904 | 14.8 |  | 4078 | 45.6 |
| Diagnosis of atrial fibrillation | 156991 | 6.7 |  | 7507 | 42.6 |  | 82959 | 3.7 |  | 2077 | 23.2 |
| Diagnosis of stroke | 124706 | 5.3 |  | 2257 | 12.8 |  | 84455 | 3.8 |  | 1026 | 11.5 |
| Diagnosis of congestive heart disease | 90788 | 3.9 |  | 12715 | 72.2 |  | 49548 | 2.2 |  | 5009 | 56.0 |
| Diagnosis of amyloidos | 1705 | 0.1 |  | 342 | 1.9 |  | 1214 | 0.1 |  | 133 | 1.5 |
| Diagnosis of systemic connective tissue disorders | 21977 | 0.9 |  | 344 | 2.0 |  | 50362 | 2.2 |  | 429 | 4.8 |
| Diagnosis of sarcoidosis | 9134 | 0.4 |  | 174 | 1.0 |  | 6587 | 0.3 |  | 83 | 0.9 |
| Diagnosis of hemochromatosis | 2498 | 0.1 |  | 27 | 0.2 |  | 1517 | 0.1 |  | 21 | 0.2 |
| Diagnosis of thyroid disorders | 22517 | 1.0 |  | 572 | 3.2 |  | 104104 | 4.6 |  | 885 | 9.9 |
| Diagnosis of chronic rheumatic heart disease | 2529 | 0.1 |  | 163 | 0.9 |  | 2632 | 0.1 |  | 103 | 1.2 |
| Diagnosis of non-rheumatic valvular heart diseases | 49608 | 2.1 |  | 2163 | 12.3 |  | 30979 | 1.4 |  | 1027 | 11.5 |
| Diagnosis of cancer | 350104 | 14.9 |  | 3751 | 21.3 |  | 357352 | 15.9 |  | 2347 | 26.3 |

| **Supplementary Table 6. The relative risk of cardiomyopathy in first-generation immigrants vs Swedish-born individuals expressed as hazard ratios (HR) with 99% confidence intervals (99% CI)** | | | | | | | |  |
| --- | --- | --- | --- | --- | --- | --- | --- | --- |
|  | Model 1 | | |  | Model 2 | | |  |
|  | HR | 99% CI | |  | HR | 99% CI | |  |
| **Men** |  |  |  |  |  |  |  |  |
| Sweden | 1 |  |  |  | 1 |  |  |  |
| All foreign-born | **0.81** | **0.77** | **0.86** |  | **0.79** | **0.75** | **0.84** |  |
| Nordic countries | **0.82** | **0.75** | **0.90** |  | 0.98 | 0.89 | 1.07 |  |
| Southern Europe | **0.50** | **0.38** | **0.66** |  | **0.58** | **0.44** | **0.77** |  |
| Western Europe | 0.88 | 0.72 | 1.08 |  | 1.07 | 0.88 | 1.31 |  |
| Eastern Europe | 0.91 | 0.77 | 1.06 |  | **0.77** | **0.66** | **0.90** |  |
| Baltic countries | 0.73 | 0.45 | 1.18 |  | 1.01 | 0.62 | 1.65 |  |
| Central Europe | 1.04 | 0.84 | 1.30 |  | 1.05 | 0.84 | 1.31 |  |
| Africa | **1.25** | **1.00** | **1.56** |  | 0.91 | 0.72 | 1.13 |  |
| Northern America | **0.45** | **0.27** | **0.76** |  | **0.48** | **0.29** | **0.81** |  |
| Latin America | **0.52** | **0.36** | **0.76** |  | **0.42** | **0.29** | **0.61** |  |
| Asia | **0.78** | **0.68** | **0.89** |  | **0.57** | **0.50** | **0.65** |  |
| Russia | 0.72 | 0.37 | 1.38 |  | 0.69 | 0.36 | 1.32 |  |
|  |  |  |  |  |  |  |  |  |
| **Women** |  |  |  |  |  |  |  |  |
| Sweden | 1 |  |  |  | 1 |  |  |  |
| All foreign-born | **0.87** | **0.80** | **0.94** |  | **0.81** | **0.75** | **0.87** |  |
| Nordic countries | **0.87** | **0.77** | **0.97** |  | 0.97 | 0.87 | 1.09 |  |
| Southern Europe | **0.58** | **0.37** | **0.90** |  | 0.64 | 0.41 | 1.00 |  |
| Western Europe | 1.01 | 0.79 | 1.30 |  | 1.23 | 0.96 | 1.58 |  |
| Eastern Europe | 0.88 | 0.70 | 1.11 |  | **0.69** | **0.54** | **0.87** |  |
| Baltic countries | 0.95 | 0.56 | 1.62 |  | 1.20 | 0.70 | 2.03 |  |
| Central Europe | 1.01 | 0.76 | 1.33 |  | 0.90 | 0.68 | 1.18 |  |
| Africa | 0.97 | 0.62 | 1.52 |  | **0.59** | **0.37** | **0.92** |  |
| Northern America | 0.58 | 0.29 | 1.13 |  | 0.62 | 0.32 | 1.22 |  |
| Latin America | 0.83 | 0.55 | 1.26 |  | **0.62** | **0.41** | **0.93** |  |
| Asia | 0.86 | 0.70 | 1.04 |  | **0.56** | **0.46** | **0.67** |  |
| Russia | 0.75 | 0.38 | 1.50 |  | 0.60 | 0.30 | 1.19 |  |
| Model 1: adjusted for age; model 2: adjusted for cancer. | | | | |  |  |  |  |

| **Supplementary Table 7. The incidence of cardiomyopathy in male and female second-generation immigrants vs native Swedes expressed as hazard ratios (HR) with 99% confidence intervals (99% CI)** | | | | | | | | |  |
| --- | --- | --- | --- | --- | --- | --- | --- | --- | --- |
|  | Model 1 | | |  | Model 2 | | |  | |
|  | HR | 99% CI | |  | HR | 99% CI | |  | |
| **Men** |  |  |  |  |  |  |  |  | |
| Sweden | 1 |  |  |  | 1 |  |  |  | |
| All foreign-born | 1.01 | 0.93 | 1.09 |  | **0.73** | **0.68** | **0.79** |  | |
| Nordic countries | **1.10** | **1.00** | **1.21** |  | **0.84** | **0.77** | **0.92** |  | |
| Southern Europe | 0.94 | 0.61 | 1.45 |  | **0.55** | **0.35** | **0.85** |  | |
| Western Europe | 0.86 | 0.67 | 1.08 |  | **0.67** | **0.53** | **0.84** |  | |
| Eastern Europe | 1.03 | 0.72 | 1.45 |  | **0.50** | **0.35** | **0.71** |  | |
| Baltic countries | 0.85 | 0.59 | 1.21 |  | 0.86 | 0.60 | 1.23 |  | |
| Central Europe | 0.80 | 0.54 | 1.16 |  | **0.54** | **0.37** | **0.79** |  | |
| Africa | 0.93 | 0.36 | 2.41 |  | 0.41 | 0.16 | 1.05 |  | |
| Northern America | 1.00 | 0.69 | 1.44 |  | 1.13 | 0.78 | 1.64 |  | |
| Latin America | 0.57 | 0.22 | 1.46 |  | **0.26** | **0.10** | **0.66** |  | |
| Asia | 0.86 | 0.58 | 1.27 |  | **0.38** | **0.26** | **0.56** |  | |
| Russia | 0.87 | 0.46 | 1.64 |  | 0.95 | 0.50 | 1.79 |  | |
|  |  |  |  |  |  |  |  |  | |
| **Women** |  |  |  |  |  |  |  |  | |
| Sweden | 1 |  |  |  | 1 |  |  |  | |
| All foreign-born | **1.02** | **0.91** | **1.14** |  | **0.73** | **0.65** | **0.82** |  | |
| Nordic countries | 1.05 | 0.92 | 1.21 |  | **0.79** | **0.69** | **0.90** |  | |
| Southern Europe | 1.15 | 0.63 | 2.08 |  | 0.64 | 0.35 | 1.16 |  | |
| Western Europe | 0.99 | 0.72 | 1.37 |  | 0.75 | 0.54 | 1.03 |  | |
| Eastern Europe | 0.72 | 0.38 | 1.34 |  | **0.34** | **0.18** | **0.63** |  | |
| Baltic countries | 1.20 | 0.78 | 1.83 |  | 1.20 | 0.78 | 1.84 |  | |
| Central Europe | 1.04 | 0.65 | 1.68 |  | 0.70 | 0.43 | 1.13 |  | |
| Africa | 1.58 | 0.54 | 4.65 |  | 0.67 | 0.23 | 1.98 |  | |
| Northern America | 1.00 | 0.60 | 1.67 |  | 1.14 | 0.68 | 1.90 |  | |
| Latin America | 0.56 | 0.14 | 2.33 |  | 0.25 | 0.06 | 1.03 |  | |
| Asia | 0.78 | 0.42 | 1.46 |  | **0.33** | **0.18** | **0.62** |  | |
| Russia | 0.66 | 0.24 | 1.81 |  | 0.72 | 0.26 | 1.97 |  | |
| Model 1: adjusted for age; model 2: adjusted for cancer. | | | | |  |  |  |  | |
